# Histamine Poisoning from Ingestion of Fish or Scombroid Syndrome

**DOI:** 10.1155/2014/482531

**Published:** 2014-12-07

**Authors:** Vincenzo Tortorella, Peppino Masciari, Mario Pezzi, Assunta Mola, Simona Paola Tiburzi, Maria Concetta Zinzi, Annamaria Scozzafava, Mario Verre

**Affiliations:** ^1^Anaesthesia and Intensive Care Unit, General Hospital “Pugliese-Ciaccio”, Viale Pio X, 88100 Catanzaro, Italy; ^2^Emergency Medicine Unit, General Hospital “Pugliese-Ciaccio”, 88100 Catanzaro, Italy

## Abstract

The scombroid poisoning is due to the ingestion of poorly preserved fish (especially tuna, sardines, and mackerel) out of the cold chain. Under the influence of the proliferation of gram negative bacteria that occurs for heating, the histidine content in the muscle of the fish is converted into histamine, by the action of the enzyme histidine decarboxylase. If the histamine is ingested in large quantities, it causes an anaphylactoid reaction with a variety of symptoms from moderate to severe to life-threating. We will describe two cases that came under our observation after consuming a meal of bluefin tuna. The diagnosis of scombroid syndrome was made on the basis of the anamnestic data and the clinical one. The rapid resolution of the signs and symptoms after treatment with histamines H1-H2 receptor blockers confirmed the suspected diagnosis.

## 1. Introduction

The scombroid syndrome is a pseudoallaergic poisoning caused by the consumption of contaminated fish belonging to the Scombridae family. It is the most common fish poisoning in Europe and in the world since this kind of fish is consumed in large quantities in all the continents: tuna, bonito, and mackerel [1].

The syndrome can also be triggered by different species from the Scombridae family. Examples include mahi-mahi (dolphin fish), sea urchins, herring, sardines, anchovies, and bluefish.

This poisoning accounts for about 5% of all reported food poisoning in the United States and about 40% of poisonings resulting from ingestion of fish [2].

It is believed that the incidence is higher because many cases are not reported since the symptoms may last for a short time.

## 2. Clinical Cases

A 32-year-old woman and her husband, 35-year-old, arrived in the emergency department of a suburban general hospital complaining of having palpitations, headache, heat all over their body, and the presence of redness especially on their face together with eyelid edema. The vital parameters of both patients showed marked hypotension (female 85/50 mmHg, man 70/35 mmHg) and tachycardia (female 105 beats/min, man 110 beats/min).

The initial treatment was the following for both patients: hydration with Ringer's lactate, methylprednisolone 2 g, and chlorpheniramine maleate 10 mg.

The male patient due to marked hypotension was treated with continuous infusion of dopamine 10 mcg/kg/min. During their stay in the emergency room the clinical conditions of both patients did not improve, and the male patient's conditions were complicated too by the onset of ventricular fibrillation. He was then promptly defibrillated, endotracheally intubated by the oral tracheal, and subjected to artificial ventilation and sedation with midazolam to fentanyl by continuous infusion.

Both patients were transferred by ambulance to our intensive care unit where they arrived three hours after their meal.

**Figure 1:**
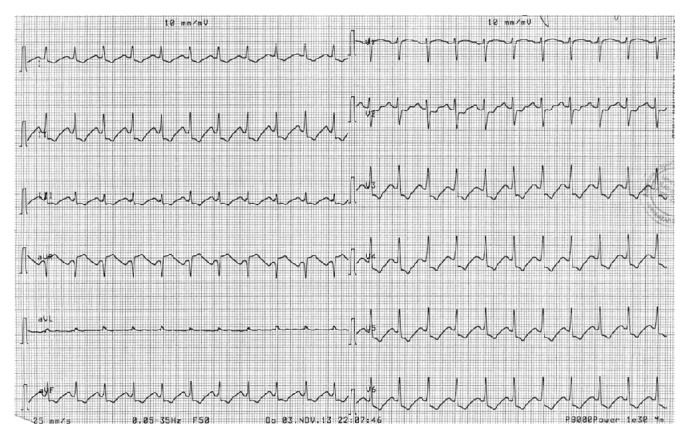
Explanation in the text.

The woman arrived conscious and cooperative. She showed a diffused erythema over her whole body, eyelid edema, and hypotension. During the clinic checkup the female patient began to have chest pain and at the ECG an underlevelment of the ST-segment became immediately evident in all precordial derivations (Figure 1). The echocardiography pointed out apical hypokinesia and apical septal hypokinesia. Immediately after administration of 50 mg of ranitidine IV we saw an immediate remission, both of the erythema and of precordial pain. The mapping changed and the underlevelment of the ST-segment disappeared.

The woman was under observation in intensive care for the next 12 hours. The treatment was carried out as follows: 150 mg of ranitidine as a continuous infusion for 12 hours, chlorpheniramine maleate 10 mg IM and Ringer lactate 1000 mL, glucose 5% solution 1000 mL, and saline solution NaCl 0,9% 1000 mL. Subsequently she was transferred to the department of emergency medicine, where she remained inpatient for another 24 hours.

**Figure 2:**
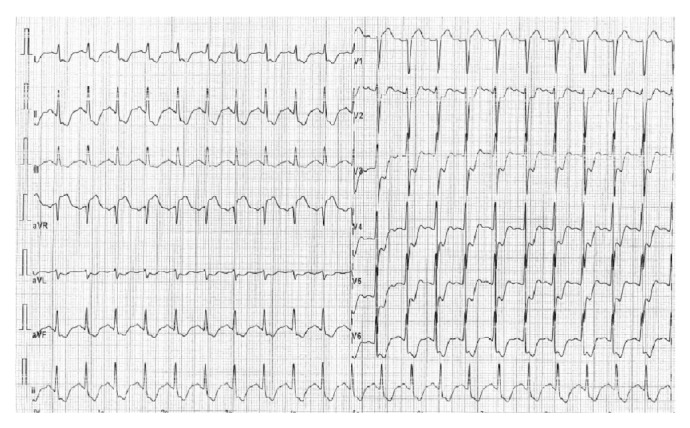
Explanation in the text.

The male patient came to our observation sedated and intubated by the oral tracheal and artificial ventilation. He showed a diffused erythema over his whole body, eyelid edema, and marked hypotension. The electrocardiogram showed a sinus tachycardia and ST-segment depression in leads bipolar and unipolar limb and precordial derivations (Figure 2).

He had the same treatment as his wife. Due to the severity of the clinical status and because of the previous ventricular fibrillation, he underwent coronary angiography, which resulted, as expected, negative (coronary arteries).

The patient was sedated and subjected to controlled ventilation until the next morning when the pharmacological sedation was suspended. When he gained consciousness and started breathing well, he was extubated and left in spontaneous breathing.

After 12 hours he was transferred to department of internal medicine and was discharged 48 hours later.

## 3. Discussion

The poisoning occurs because of an inadequate cooling and poor preservation of the fish caught. It is found mainly in warm or temperate waters particularly in countries lacking infrastructure for the preservation and storage of food. The bacterial spoilage and the production of histamine can occur at any stage of the food chain (fishing and fish landing, processing, distribution systems, or as caterers or home). The muscle mass of the fish is characterized by red looking meat and by large quantities of histidine. A typical example is the “bluefin tuna” that is used to prepare sashimi or sushi.

The high content of histidine free in the tissues of fish of the family Scombridae is typical of migratory pelagic species such as mackerel, tuna, and yellow fin tuna. The histidine can have a buffer effect, protecting the tissues from the sudden increase of lactic acid [3].

The metabolism of histidine follows basically two ways: the major route of catabolism of histidine passes through its transformation to glutamic acid, which begins with the degradation of histidine to urocanic acid by action of the enzyme histidase. The glutamate product is converted to alpha-ketoglutarate, which is an intermediate in the citric acid cycle (Krebs cycle). The second is the decarboxylation (loss COO-) for action of the enzyme histidine decarboxylase with formation of histamine.

The range of optimum pH for the activity of histidine decarboxylase is 2.5 to 6.5. The pH of the fresh scombroids ranges from 5.5 to 6.5; this level of mild acidity favors the production of histamine from the bacterial decarboxylase [3].

The free histidine is metabolized during the heating and the deterioration of the meat to histamine, histamine phosphate, histamine hydrochloride, and saurina by the enzyme histidine decarboxylase produced by the bacteria present in the fish.

The process of decarboxylation is induced mainly by enzymes produced by gram negative enteric bacteria (e.g.,* Morganella morganii*,* Escherichia coli*,* Klebsiella* spp., and* Pseudomonas aeruginosa*) that are found in the intestine or in the skin of the fish [4]. These bacteria are commonly found in the marine environmentand are found naturally in the intestine and gills of the fish, without causing them injury or disease. To prevent poisoning the fish needs to be continuously iced or refrigerated at less than or equal to 32 degrees F (0 degrees C) from the time the fish is caught until it is prepared for consumption [5]. If the preservation methods are not good, the degradation of histidine to histamine can be caused by the bacteria that grow due to the heat of the sun (the* Achromobacter histamineum* is the main species in question) [1].

The oral administration of histamine pure does not cause systemic effects, since it is inactivated in the intestine prior to entering the portal circulation, converted from enteric flora. Histamine is also “fixed” by the intestinal mucins [6].

To explain the massive absorption of histamine in scombroid syndrome, the presence of enhancers is assumed. Diamines can enhance the toxic action of histamine facilitating its passage through the intestinal wall into the circulation.

The action of enhancers goes to interfere with the protective action of intestinal mucins, which “bind” histamine, and it was suggested that this binding is essential for preventing enteral absorption; strengthening would occur due to the breaking of the bond and increase absorption [7].

Histamine is therefore not present in the fish alive, but it is produced after his death, when the defense mechanisms no longer inhibit bacterial growth. The conversion of histidine to histamine occurs in the early stage of the deterioration of the fish when it is still apparently edible [5].

The fish may have a sharp smell and peppery or metallic taste even if this does not mean the fish is not toxic if it does not have these characteristics.

No method of preparation or cooking can eliminate the histamine present. Cooking destroys only the bacteria that produce histamine but not the histamine already produced because it is thermostable.

People who ingest the rotten fish can have a pseudoallergic reaction triggered by histamine.

The FDA considers a toxic value of histamine higher than 50 mg/100 g of tuna [4].

The normal concentration is less than 0.1 mg/100 g of fish [8].

In a review of the literature on 250 cases of poisoning [9] values of histamine at risk for intoxication have been suggested as follows:

1. less than 5 mg of histamine/100 g of fish: considered safe,
2. from 5 to 10 mg of histamine/100 g of fish: possibly toxic,
3. 20–100 mg of histamine/100 g of fish: probably toxic,
4. value higher than 100 mg of histamine/100 g of fish: toxic.

There is a considerable individual variation in sensitivity towards scombroid poisoning.

Most individuals do not develop the disease even with concentrations of histamine of 100 mg/100 g of fish. However, cases of poisoning have been reported in susceptible individuals even with concentrations of histamine of 20 mg/100 g or less [10].

The pathogenesis of the syndrome is not completely clear yet. Several hypotheses are reported in a review by Hungerford [11]:

1. the strengthening of histamine toxicity by other toxic compounds found in fish such as cadaverine which would result in an increased absorption of histamine through the intestines;
2. inhibition of toxicity-enhancing action of histamine by inhibitors of histamine-metabolizing enzymes: diamine oxidase (DAO) and histamine N-methyl transferase (HNMT); the cadaverine produced clear increases in histamine levels 2 to 5 times;
3. Mast cell degranulation: it is said that there may be a scombrotoxin linked to the spoiled fish able to cause mast cell degranulation;
4. other agonists of histamine of the receptors with total histamine similar activity; specific substances in scombroid syndrome have not been found so far;
5. histamine intolerance a condition that describes the high sensitivity to histamine in a diet including food and wine rich in histamine; it is a metabolic disorder resulting from the imbalance between the histamine eaten and the ability of the metabolism by one's body; so that is how we can explain the difference in individual susceptibility to histamine present in the decomposed fish.

The clinical manifestations of the syndrome can appear from five minutes to several hours after ingestion, and it depends on the concentration of histamine in the fish [12]. The effect is exerted by binding to receptors in the cell membrane of the respiratory, cardiovascular, gastrointestinal, and immune system.

A review on 46 patients [13] showed the incidence of dermatologic manifestations in 82.2%, gastrointestinal in 37%, neurological in 34.7%, respiratory in 17.4%, general (malaise and weakness) in 4.3%, and cardiovascular in 37.8% of the intoxicated patients.

The symptoms most frequently reported are [12] as follows.

- Skin: rash, urticaria, localized swelling, and erythema on one's face, neck, and trunk.
- Gastrointestinal apparatus: nausea, vomiting, diarrhea, epigastric pain, and cramping.
- Circulatory system: conjunctival injection, hypotension or hypertension, tachycardia, and palpitations, up to shock.
- Nervous system: headache, tingling, cramps, feeling of warmth around the mouth, and loss of sight.
- Respiratory system: bronchoconstriction and respiratory distress.

The poisoning can be serious and even fatal if not treated in time. Several severe clinical cases of particular complexity have been described with serious cardiac complications [14–16] or related arterial hypotension [17, 18]. The histamine produces vasodilation on the peripheral circulation, through the release of nitric oxide, increased capillary permeability with consequent possible development of besides urticaria, headache, erythema, and even systemic hypotension. It causes vasoconstriction on the circulation coronary instead. Moreover it causes an increased atrial and ventricular muscle contractility through the promotion of the influx of calcium into the cells on one's heart, increased heart rate for potentiating action on the automatism of the sinoatrial node with potential arrhythmic risk for the increase of automaticity of atrial cells, Purkinje and ventricular.

The treatment for mild or moderate cases is based on the use of antihistamines, both anti-H1 (e.g., diphenhydramine every 6 hours as needed, 25 to 100 mg in adults, 0.5–1.5 mg/kg in children) and anti-H2 (e.g., cimetidine, ranitidine, and famotidine) [19].

In severe cases, antihistamines are administered intravenously, both anti-H1 (e.g., diphenhydramine every 6 hours as needed, 25 to 100 mg in adults, 0.5–1.5 mg/kg in children) and anti-H2 (e.g., cimetidine, ranitidine, and famotidine), isotonic crystalloid fluids if the patient is hypotensive. If there is edema in the respiratory tract, hypotension, and bronchospasm, epinephrine (adults: 0.3 mg IM; children: 0.01 mg/kg IM) and corticosteroids (prednisone 60 mg for adults orally or methylprednisolone sodium succinate 125 mg IV) are administered [20]. In case of bronchospasm beta agonists are used (e.g., salbutamol) by inhalation.

## 4. Conclusions

The rapid cooling of the fish caught and a rigorous maintenance of the cold chain following the guidelines of the FDA [8] until the consumption of the product are essential to prevent the degradation of the meat, bacterial proliferation, activation of the enzyme histidine decarboxylase, the conversion of histidine to histamine, and the development of anaphylactoid reaction to histamine following ingestion of the fish. The diagnosis is made primarily on the basis of clinical history, symptomatology, and epidemiological studies. The patient may report that he or she tasted something spicy and metallic in the fish. The differential diagnosis is between the histamine poisoning and the allergic reactions.

If the syndrome develops, an immediate recognition and treatment with antihistamines, anti H1 and anti-H2, are required.
